# Regulation of angiopoietin‐like protein 4 production during and after exercise

**DOI:** 10.14814/phy2.12109

**Published:** 2014-08-19

**Authors:** Frode Norheim, Marit Hjorth, Torgrim M. Langleite, Sindre Lee, Torgeir Holen, Christian Bindesbøll, Hans K. Stadheim, Hanne L. Gulseth, Kåre I. Birkeland, Anders Kielland, Jørgen Jensen, Knut T. Dalen, Christian A. Drevon

**Affiliations:** 1 Department of Nutrition, Faculty of Medicine, Institute of Basic Medical Sciences, University of Oslo, Oslo, Norway; 2 Department of Physical Performance, Norwegian School of Sport Sciences, Oslo, Norway; 3 Department of Endocrinology, Morbid Obesity and Preventive Medicine, Oslo University Hospital, Oslo, Norway; 4 Faculty of medicine, University of Oslo, Oslo, Norway

**Keywords:** Adipose tissue, ANGPTL4, exercise, skeletal muscle

## Abstract

Angiopoietin‐like protein 4 (ANGPTL4) may regulate lipoprotein lipase‐dependent plasma clearance of triacylglycerol from skeletal muscle during exercise. The aim of this study was to examine the importance of muscle in regulating ANGPTL4 in response to exercise. We sampled muscle biopsies and serum before, immediately after, and 2 h after 45 min of ergometer cycling. Sampling was done before and after a 12‐week training intervention in controls and dysglycemic subjects. Moreover, fat biopsies were taken before and after the training intervention. The regulation of ANGPTL4 was also investigated in several tissues of exercising mice, and in cultured myotubes. ANGPTL4 levels in serum and expression in muscle were highest 2 h after exercise in both groups. Whereas ANGPTL4 was higher in muscle of exercising controls as compared to dysglycemic subjects, the opposite was observed in serum. In exercising mice, *Angptl4 *mRNA showed both higher basal expression and induction in liver compared to muscle. *Angptl4 *mRNA was much higher in adipose tissue than muscle and was also induced by exercise. We observed two mRNA isoforms of *ANGPTL4* in muscle and fat in humans. Both were induced by exercise in muscle; one isoform was expressed 5‐ to 10‐fold higher than the other. Studies in mice and cultured myotubes showed that both fatty acids and cortisol have the potential to increase *ANGPTL4* expression in muscle during exercise. In conclusion, *ANGPTL4* is markedly induced in muscle in response to exercise. However, liver and adipose tissue may contribute more than muscle to the exercise‐induced increase in circulating ANGPTL4.

## Abstract (precis)

The Production of ANGPTL4 is markedly induced in skeletal muscle in response to exercise. However, liver and adipose tissue may contribute more than skeletal muscle to the exercise‐induced increase in circulatory ANGPTL4.

## Introduction

During the last decade, it has become apparent that skeletal muscle is a major endocrine organ (Pedersen and Febbraio 2012). Skeletal muscle can synthesize and secrete several hundred proteins (Bortoluzzi et al. 2006; Henningsen et al. 2010; Norheim et al. 2011). Proteins that are expressed, synthesized, released by myofibers, and exert either paracrine or endocrine effects, are classified as “myokines” (Pedersen and Febbraio 2012). Although skeletal muscle hypertrophy and regeneration may enhance myokine secretion for a long period (Ambrosio et al. 2009; Norheim et al. 2011; Shan et al. 2013), acute muscle fiber contraction can induce myokine secretion rapidly, as shown for IL‐6 (Pedersen and Febbraio 2012).

ANGPTL4 is a multifunctional signal protein synthesized by most tissues (Grootaert et al. 2012). It is involved in regulation of angiogenesis, glucose and lipid metabolism, and cell differentiation (Grootaert et al. 2012). Several studies show that ANGPTL4 inhibits the activity of lipoprotein lipase (LPL), which is responsible for hydrolysis of plasma triacylglycerols (TG) to monoacylglycerols and free fatty acids (FFAs) (Yoshida et al. 2002; Mandard et al. 2006; Desai et al. 2007; Grootaert et al. 2012). ANGPTL4 may also stimulate adipose tissue lipolysis, and thereby release of glycerol and FFAs to the circulation (Yoshida et al. 2002; Mandard et al. 2006). Thus, the net effect of ANGPTL4 will represent a shift of FA oxidation derived from lipoproteins toward FAs originating from adipose tissue. Furthermore, ANGPTL4 is positively associated with body fat mass (Smart‐Halajko et al. 2010).

Expression of *ANGPTL4* is increased via different peroxisome proliferator‐activated receptors (PPARs) and the glucocorticoid receptor (GR) in hepatocytes and adipocytes (Kersten et al. 2000; Koliwad et al. 2009; Grootaert et al. 2012). Studies on cultured human myotubes show that secretion of ANGPTL4 is stimulated by fatty acids (FAs) as well as the PPAR*δ*‐specific activator GW501516 (Kersten et al. 2009; Staiger et al. 2009; Robciuc et al. 2012). The impact of exercise on *ANGPTL4* expression during exercise is not fully understood. Previous studies suggest that ANGPTL4 is an exercise‐responsive myokine regulated by circulating factors (Kersten et al. 2009; Catoire et al. 2014). Kersten et al. (2009) reported that the plasma concentration of ANGPTL4 after endurance activity is less enhanced in subjects given oral glucose, and Catoire et al. (2014) demonstrated in a human one‐legged exercise study a stronger induction of *ANGPTL4* mRNA in the resting leg as compared to the exercising leg. The inhibitory effect of glucose on *ANGPTL4* transcription is probably caused by increased release of insulin, causing suppression of lipolysis and reduced plasma FFA concentration (Kersten et al. 2009; Catoire et al. 2014). Furthermore, the stimulatory effect of plasma FFA on skeletal muscle ANGPTL4 expression might be counteracted by the activation of AMPK‐activated kinase (AMPK) in exercising muscle. To our knowledge, it is not known if ANGPTL4 produced in skeletal muscle mainly act on the local tissue or whether the protein also convey an endocrine effect.

The main aim of this study was to examine the importance of skeletal muscle in regulating circulating ANGPTL4. We also investigated if transcription of ANGPTL4 in skeletal muscle is regulated solely via PPAR*δ* or if additional exercise‐related factors such as glucocorticoids may play a role.

## Materials and Methods

### Ethical approval

The study adhered to the Declaration of Helsinki and was approved by the National Regional Committee for Medical and Health Research Ethics North, Tromsø, Oslo, Norway. The study was registered with the US National Library of Medicine Clinical Trials registry (NCT01803568). Written informed consent was obtained from all participants prior to any study‐related procedure.

### Strength and endurance training intervention

Healthy and physically inactive men (40–65 years) were recruited and divided into two groups; controls with normal weight (23.5 ± 2.0 kg/m^2^) and normal fasting and 2‐h serum glucose levels (*n* = 13) or overweight (29.0 ± 2.4 kg/m^2^) with abnormal glucose metabolism (dysglycemic group, *n* = 13). Abnormal glucose metabolism was defined as fasting glucose ≥5.6 mmol/L and/or impaired glucose tolerance (2‐h serum glucose ≥7.8 mmol/L). The participants were subjected to a combined strength and endurance training program for 12 weeks, including two endurance bicycle sessions (60 min) and two whole‐body strength‐training sessions (60 min) per week. Each endurance session started with a 10‐min warm‐up at three different workloads, corresponding to 50% (4 min), 55% (3 min), and 60% (3 min) of VO_2_max. A 45‐min bicycle session at 70% of VO_2_max was performed before and after the 12‐week training period as an acute work challenge.

A carbohydrate‐rich meal was provided 90–120 min before the exercise test included bread, cheese, jam, and apple juice, providing 23% of estimated total daily energy expenditure, on average 2475 KJ. Tests were performed in the morning, so the standardized meal was the only intake after overnight fast. A few subjects were tested in the afternoon (at the same time of day before as well as after 12 weeks of training) and had the standardized meal as their only intake during the last 4–5 h. Water could be consumed freely.

### Blood and tissue sampling

Blood and muscle samples were taken before, directly after, and 2 h after the 70% of VO_2_max bicycle test, before as well as after 12 weeks of training. Muscle biopsies were lacking for one subject at 2 h post exercise, before as well as after 12 weeks of training. Blood samples were taken by standard antecubital venous puncture. A single subcutaneous adipose tissue biopsy in the periumbilical region was taken ~30 min after the bicycle session, before as well as after 12 weeks of training. Subcutaneous adipose biopsies were obtained from 13 controls and 11 dysglycemic subjects before as well as after 12 weeks of training.

Biopsies from *m. vastus lateralis* were immediately transferred to RNA‐later (Qiagen, Limburg, the Netherlands), kept overnight at 4°C, and transferred to −80°C. Subcutaneous biopsies were frozen immediately in liquid nitrogen and stored at −80°C until further processing. Serum and EDTA plasma were stored at −80°C until further analysis.

### Serum and plasma analyses

Serum samples of ANGPTL4 (Catalog # RAB0017, Sigma‐Aldrich, St. Louis, MO) and cortisol (Catalog # ab108665, Abcam, Cambridge, UK) were measured in duplicates using enzyme‐linked immunesorbent assays according to the manufacture's protocol. Optical density was determined using a microplate reader (Titertec Multiscan Plus; EFLAB, Helsinki, Finland) set to 450 nm. Standard curves for ANGPTL4 and cortisol were generated with a 4 parameters logistics curve‐fitting method (MyAssays.com). One subject with extremely high levels of ANGPTL4 in serum (80–225 ng/mL) was excluded from the ANGPTL4 analysis. FFA plasma levels were determined using a Maxmat PL multianalyzer (Maxmat, France) with reagents (Catalog # D07940/D07950, DIALAB, Wiener Neudorf, Austria).

### Cell culture

Primary human myoblasts from *m. obliquus internus abdominis* of healthy kidney donors were isolated (Haugen et al. 2010). Myoblasts at passage 5 were proliferated on collagen I‐coated dishes in DMEM/Ham's F12 1:1 (Life Technologies, Grand Island, NY) containing glutamax (Life Technologies), 50 U/mL penicillin, 50 g/mL streptomycin, 5 mmol/L glucose, 10% FBS, 70 pM insulin, 10 ng/mL epidermal growth factor, and 2 ng/mL basic fibroblast growth factor (Sigma‐Aldrich). When the cultures were near confluency, the myoblasts were differentiated into multinucleated myotubes by changing medium to DMEM/Ham's F12 1:1 (5 mmol/L glucose) containing glutamax, 50 U/mL penicillin, 50 *μ*g/mL streptomycin, and 2% horse serum (Sigma‐Aldrich). After 6 days of differentiation, myotubes were incubated with 0, 0.1, 0.5, and 2 *μ*mol/L of dexamethasone (Sigma‐Aldrich). All biopsies were obtained with informed written consent and approved by the National Committee for Research Ethics, Oslo, Norway.

Mouse muscle‐derived C2C12 cells were transfected with PPAR isoforms and cultured with FAs and PPAR (ant‐) agonists (Bindesboll et al. 2013). The PPAR (ant‐) agonists WY‐14643 (PPAR*α* agonist), GW6471 (PPAR*α* antagonist), GSK0660 (PPAR*δ* antagonist), GW9662 (PPAR*γ* antagonist), and FAs were obtained from Sigma‐Aldrich. GW501516 (PPAR*δ* agonist) and rosiglitazone (Rosi) (PPAR*γ* agonist) were from Enzo Life Sciences.

### Mouse experiments

All animals used were approved and registered by the Norwegian Animal Research Authority. C57BL/6 male mice were housed in a temperature‐controlled (22°C) facility with a strict 12‐h light/dark cycle. Mice were anesthetized with 2.5% isoflurane before EDTA plasma was collected, and euthanized by cervical dislocation prior to harvest of muscles. Plasma and tissue samples were frozen immediately on dry ice and in liquid nitrogen, respectively, and stored at −80°C.

Six‐month‐old mice were exposed to treadmill exercise (TSE Systems, Germany) prior to sampling of EDTA plasma, calf muscles (gastrocnemius and soleus), perirenal fat, and liver. To avoid stress‐related responses, the mice were gradually habituated to treadmill running before a final strenuous exercise session. The first session included 2‐min free walk on the treadmill with low speed. The next six sessions followed an identical warm‐up pattern including a speed of 0.10 m/s for 2 min and 0.15 m/s for 8 min. In session 3–7, the mice additionally ran at 0.25 m/s for 5, 10, 15, 30, and 45 min. The last session ended with an additional final graded increase in speed from 0.25 to 0.35 m/s for a 5‐min period. A platform placed behind the running band enabled the mice to rest from running. Running was encouraged by up to five gentle electrical shocks (0.3 mA) within one exercise session. Mice that after five shocks still sat on the platform were defined as exhausted and removed from the treadmill. All exercise sessions were performed between 2 pm and 4 pm with no food withdrawal prior to exercise. Blood and muscle samples were taken immediately after the final exercise. Control mice (*n* = 8) were housed in similar cages as the trained mice (*n* = 8) and were handled equally except for the exercise regimen.

Four‐month‐old mice on a standard chow diet were given intragastric gavage of 0.5% carboxymethylcellulose (CMC) (Sigma # C4888) or 300 *μ*L GW501516 (150 *μ*L solved in 0.5% CMC; 5 mg/kg) (Bindesboll et al. 2013). Mice were gavaged 36 and 12 h before being euthanized at the onset of the light cycle (*n* = 4–6 in each group).

### RNA isolation

Total RNA was isolated from cultured human and murine myotubes as described previously (Haugen et al. 2010; Bindesboll et al. 2013). Frozen human or mouse muscle biopsies were crushed to powder in a liquid nitrogen‐cooled mortar using a pestle. One‐mL QIAzol Lysis Reagent (Qiagen) was added to muscle tissue powder, perirenal fat or liver tissue, and the samples were homogenized using TissueRuptor (Qiagen) at full speed twice for 15 sec. Total RNA was then isolated by miRNeasy Mini Kit (Qiagen).

### Reverse transcription–polymerase chain reaction

Using High‐Capacity cDNA Reverse Transcription Kit (Applied Biosystems, Foster City, CA), 1000 ng of total RNA from human and mouse tissue samples or cells was converted into cDNA. The cDNA reaction mixture was diluted in water and an equivalent of 25 and 50 ng was analyzed in each sample in the mouse and human tissues, respectively. Quantitative real‐time PCR was performed in the 96‐well format using a 7900HT Fast instrument and the SDS 2.3 software (Applied Biosystems) (Haugen et al. 2010). Predesigned commercial primers and probe sets (TaqMan assays, Applied Biosystems) were used to analyze mRNA levels of *ANGPTL4* (Hs00401006_m1), *Angptl4* (Mm00480431_m1), beta‐2 microglobulin (*B2M*, Hs00984230_m1), large ribosomal protein P0 (*RPLP0*, Hs99999902_m1), and TATA box binding protein (*Tbp*, Mm00446973_m1). Relative target mRNA expression levels were calculated as 2^−ΔCt^ by normalizing to *B2M* and *RPLP0* in humans and *TBP* in mice.

### High‐throughput mRNA sequencing

All mRNA samples were deep sequenced using the Illumina HiSeq 2000 system with multiplexed design. Illumina HiSeq RTA (real‐time analysis) v1.17.21.3 was used for real‐time analysis during the sequencing run. Reads passing Illumina's recommended parameters were demultiplexed using CASAVA v1.8.2. For prealignment quality checks we used the software FastQC (http://www.bioinformatics.babraham.ac.uk/projects/fastqc/). The mean library size was 44.1 million unstranded single‐ended reads with no difference between groups or time points. Reads alignment was done using Tophat v2.0.8 (Kim et al. 2013), Samtools v0.1.18 (Li et al. 2009), and Bowtie v2.1.0 (Langmead et al. 2009) with default settings against the UCSC hg19 annotated transcriptome and genome dated 14th of May 2013. Postalignment quality checks were done using the Integrative Genome Viewer 2.3 (Robinson et al. 2011; Thorvaldsdottir et al. 2013) and BEDtools v2.19.1 (Quinlan and Hall 2010). Reads counted by gene feature were done using the intersection strict mode in HTSeq 0.6.1 (Anders et al. 2014). For differential gene expression analyses *ANGPTL4, LPL, PDK4, PPARD,* and *SLC22A5* were chosen a priori. edgeR v3.4.2 (Robinson et al. 2010) was used to calculate normalized gene expression levels in Counts Per Million reads (CPM) and statistical significance. Filtering strategies, quality check, and generalized linear model construction were done in R v3.0.3 following the developers' recommendations. For transcript‐specific isoforms differential expression analyses of the a priori chosen genes, we used Cuffdiff v2.1.1 (Trapnell et al. 2012) with default settings and no novel discovery on the Tophat v2.0.8 constructed BAM files. Reads were counted by Cufflinks v2.1.1 (Trapnell et al. 2012) by isoform feature in the general feature format annotation file for hg19 before statistical calculations were performed in Cuffdiff. The isoform‐normalized expression values are presented as reads per kilobase per million mapped reads (RPKM) to compensate for different transcript isoforms length.

### Statistical analyses

Statistical analyses were performed using Microsoft Excel and SPSS 20.0 software (IBM, New York, NY). Effect measures are presented as means ± standard error of the means (SEM) or means ± standard deviation. Statistical evaluation was done by Student's *t*‐tests for paired or unpaired observations. Statistical calculations of *ANGPTL4* mRNA isoforms were performed by Cuffdiff.

## Results

### Acute exercise tended to increase muscle *ANGPTL4* mRNA more in the controls compared to the dysglycemic subjects

**Figure 1.**
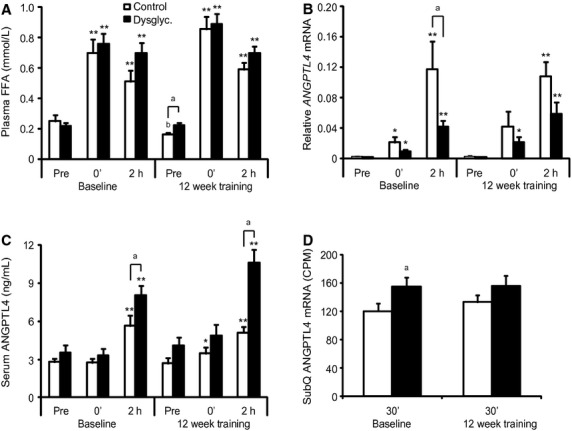
Acute exercise induces ANGPTL4 differently in muscle and serum of healthy controls and dysglycemic subjects. Changes in *m. vastus lateralis* and subcutaneous fat mRNA and serum concentration of ANGPTL4 in healthy and dysglycemic men in response to acute and chronic exercise (*n* = 26). Samples were obtained before (Pre), immediately after (0′), and 2 h after exercise (2 h) of 45‐min ergometer cycling (70% VO_2_max) at baseline and after 12 weeks of training. Muscle biopsies were processed for mRNA expression analysis by quantitative RT‐PCR and mRNA sequencing (*n* = 13 at pre and 0′, and *n* = 12 at 2 h in the controls). (A) Plasma levels of FFA; (B) Skeletal muscle *ANGPTL4 *mRNA expression using RT‐PCR; (C) Serum levels of ANGPTL4 (*n* = 12 controls, *n* = 13 dysglycemic subjects); (D) Subcutaneous fat *ANGPTL4 *mRNA expression using mRNA sequencing (*n* = 13 controls, *n* = 11 dysglycemic subjects). All quantitative RT‐PCR expression data were normalized to *B2M*. The gene expression levels of *ANGPTL4* obtained from the mRNA sequencing dataset were expressed in counts per million (CPM). Bars depict means ± SEM *P < 0.05 and **P < 0.01 between preexercise values and immediately after (0′) or 2 h post exercise. ^a^*P* < 0.05 as compared to the control group at the same sampling time point. ^b^*P *< 0.05 as compared to the same sampling point before 12 weeks of training. Student's *t*‐test was used for single comparisons.

To test the effect of acute as well as chronic exercise in healthy subjects, we obtained muscle biopsies and serum samples, before, immediately after, and 2 h post exercise of 45‐min ergometer cycling (70% VO_2max_) at baseline and after completion of a 12‐week training regime. Both healthy normal weight controls (*n* = 13) and overweight dysglycemic subjects (*n* = 13) participated in the training intervention. Twelve weeks of training significantly increased aerobic capacity (VO_2_max) by approximately 17% and insulin sensitivity as measured by the glucose infusion rate during euglycemic hyperinsulinemic clamp by approximately 30% in both groups (T. M. Langleite, unpubl. data, 2014). Exercise may enhance *ANGPTL4* mRNA expression in muscle via elevated plasma FFAs (Catoire et al. 2014). Circulating FFAs increased in response to acute exercise and showed the highest concentration immediately after the acute workload both at baseline and after 12 weeks of training (Fig. 1A). In skeletal muscle, on the other hand, *ANGPTL4* mRNA expression was highest 2 h post exercise (Fig. 1B). The *ANGPTL4* mRNA levels were significantly higher in the control group compared the dysglycemic group 2 h post exercise at baseline (Fig. 1B). Alternative splicing can make several *ANGPTL4* mRNA isoforms. We performed mRNA sequencing on muscle, which revealed the expression of two isoforms (Table 1). Both isoforms were regulated by exercise and the full‐length isoform (NM_139314) was 5‐ to 10‐fold higher expressed compared to the isoform lacking exon 4 (NM_001039667). At baseline, the full‐length *ANGPTL4* isoform was significantly higher expressed in the controls compared to the dysglycemic subjects 2 h post exercise (Table 1), which is in accordance with the qPCR data (Fig. 1B).

**Table 1.** Changes in muscle *ANGPTL4* mRNA isoforms in response to acute and chronic exercise

Group	Isoform	Baseline Pre	0′	2 h	12 weeks Pre	0′	2 h
Control	NM_001039667	0.047	0.689^*^	0.443	0.110	0.323	0.825^*^
Dysglycemic	NM_001039667	0.150	0.361	0.937^*^	0.057	0.934^*^	0.408
Control	NM_139314	0.496	2.587^**^	11.473^**^	0.449	6.291^**^^b^>	11.625^**^
Dysglycemic	NM_139314	0.347	2.072^*^	8.007^**^^aa^	0.465	4.903^**^^b^	9.218^**^

The isoform‐normalized expression values are presented as reads per kilobase per million mapped reads and measured before (Pre), immediately after (0′), and 2 h after exercise (2 h) of 45‐min ergometer cycling (70% VO_2_max) at baseline and after 12 weeks of training.

**P < 0.01 and *P < 0.05 as compared to before (pre) acute exercise.

^aa^*P* < 0.01 as compared to the control group at the same sampling time point.

^b^*P* < 0.05 as compared to the sampling time point before 12 weeks training.

### ANGPTL4 serum concentration was higher in dysglycemic subjects compared to controls after acute exercise

Using a polyclonal antibody raised against human ANGPTL4 residues 26–406, we measured the serum levels of ANGPTL4. Circulating ANGPTL4 showed no change immediately after acute exercise in either group at baseline but was slightly increased acutely at 12 weeks in the controls (Fig. 1C). Two hours postexercise ANGPTL4 levels increased approximately two‐ and three‐fold as compared to before acute exercise in controls and dysglycemic subjects, respectively (Fig. 1C). The absolute serum levels of ANGPTL4 were significantly higher in the dysglycemic group compared to the controls 2 h post exercise at baseline as well as after 12 weeks of training (Fig. 1C), and the relative increase from before acute exercise to after 2 h rest was significantly higher in the dysglycemic subjects than the controls after 12 weeks of training (*P* = 0.03). We observed no effect of 12 weeks of training on ANGPTL4 levels at rest before acute exercise in skeletal muscle mRNA (Fig. 1A) or in serum protein (Fig. 1C).

Because of the discrepancy in ANGPTL4 regulation between the groups in skeletal muscle (Fig. 1A**)** and serum (Fig. 1C) in response to acute exercise, we investigated the *ANGPTL4* mRNA expression in subcutaneous fat. The *ANGPTL4* mRNA level was significantly higher in the dysglycemic subjects compared to the controls at baseline in adipose tissue biopsies harvested about 30 min after acute exercise (Fig 1D). Furthermore, we identified the same two *ANGPTL4* isoforms in adipose tissue as observed in muscle, and the full‐length isoform (NM_139314) was significantly higher expressed in the dysglycemic subjects at baseline (Table 2). *ANGPTL4* mRNA expression of both isoforms were markedly higher in adipose tissue (Table 2) than in muscle (Table 1). The finding of a discrepancy in ANGPTL4 regulation between the groups in skeletal muscle and serum in response to acute exercise and the fact that *ANGPTL4* mRNA level is markedly higher in adipose tissue suggest that the ANGPTL4 produced in skeletal muscle during exercise has only a minor influence on serum levels.

**Table 2.** Subcutaneous *ANGPTL4* mRNA isoforms in response to chronic training

Group	Isoform	Baseline	12 weeks
Control	NM_001039667	12.4	12.8
Dysglycemic	NM_001039667	19.0	19.8
Control	NM_139314	107.7	119.4
Dysglycemic	NM_139314	138.7^aa^	138.4

The isoform‐normalized expression values are presented as reads per kilobase per million mapped reads and measured ~30 min after acute exercise at baseline and after 12 weeks of training.

^aa^*P* < 0.01 as compared the control group at the same sampling time point.

### Acute exercise increases mRNA expression of LPL and PPAR*δ* target genes

**Figure 2.**
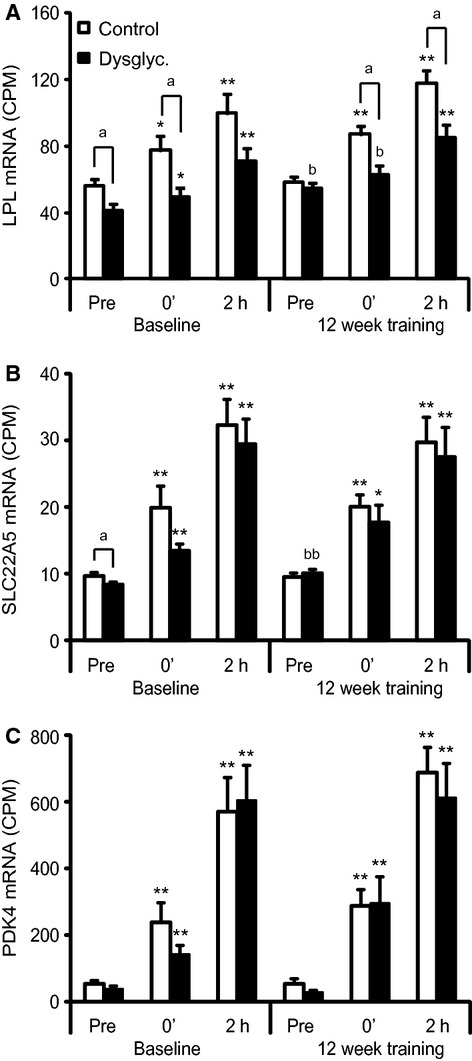
Acute exercise induces *LPL*,* SLC22A5,* and *PDK4 *mRNA levels in healthy controls and dysglycemic subjects. Changes in *m. vastus lateralis *mRNA of *LPL*,* SLC22A5,* and *PDK4* in healthy and dysglycemic men in response to acute and chronic exercise. Samples were obtained before (Pre), immediately after (0′), and 2 h after exercise (2 h) of 45‐min cycling (70% VO_2_max) at baseline and after 12 weeks of training (*n* = 26). The muscle biopsies were processed for mRNA expression analysis by mRNA sequencing (*n* = 13 at pre and 0′, and *n* = 12 at 2 h in the controls). (A) *LPL*; (B) *SLC22A5*; (C) *PDK4*. Gene expression levels obtained from the mRNA sequencing data were expressed in counts per million (CPM). Bars depict means ± SEM *P < 0.05 and **P < 0.01 between preexercise values and immediately after (0′) or 2 h post exercise. ^a^*P *< 0.05 as compared to the control group at the same sampling time point. ^b^*P *< 0.05 and ^bb^*P *< 0.01 as compared to the same sampling point before 12 weeks of training. Student's *t*‐test was used for single comparisons.

Because ANGPTL4 is a target of PPAR*δ* in skeletal muscle cells and is suggested to play an important role as an inhibitor of the enzyme LPL (Catoire et al. 2014), we investigated the mRNA expression of *LPL* and the PPAR*δ* targets *SLC22A5* (solute carrier family 22 [organic cation/carnitine transporter], member 5) and *PDK4* (Pyruvate dehydrogenase lipoamide kinase isozyme 4) in skeletal muscle. Both *LPL* (Fig 2A), *SLC22A5* (Fig. 2B) and *PDK4* (Fig. 2C) expression increased in response to acute exercise with the highest mRNA levels 2 h post exercise. The absolute mRNA expression of *LPL* was higher in the controls as compared to the dysglycemic group at most time points (Fig. 2A).

### Fatty acids stimulate *Angptl4* expression via PPAR*δ* in vivo

**Figure 3.**
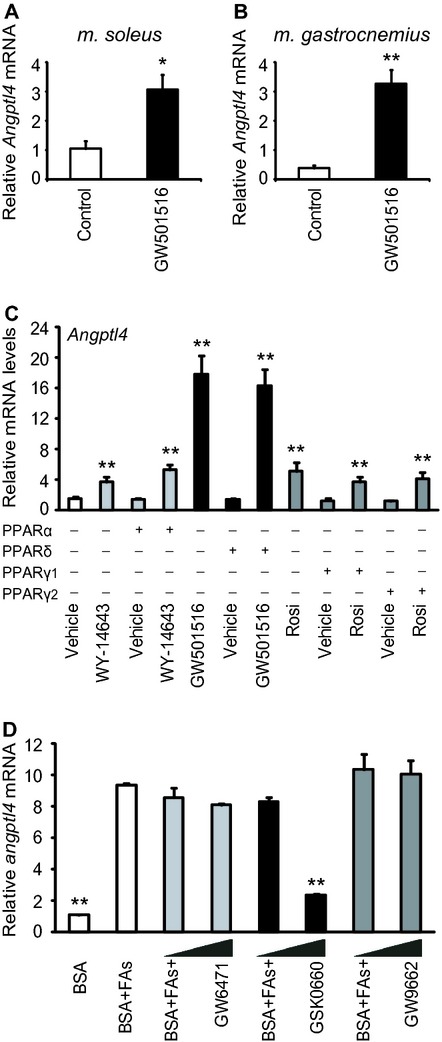
PPAR*δ* activates transcription of *Angptl4* in cultured murine myotubes and skeletal muscle. (A,B) Mice were gavaged twice (36 and 12 h before being euthanized) with vehicle (0.5% CMC) or GW501516 (5 mg/kg). (A) *Angptl4 *mRNA expression in soleus muscle; (B) *Angptl4 *mRNA expression in gastrocnemius muscle. (C) C2C12 cells were transfected with pcDNA3 vector (control) or different pcDNA3‐PPAR expression vectors at day 0, differentiated for 3 days, and stimulated with selective PPAR activators for 24 h. Activators of PPAR*α* (WY‐14643; 10 *μ*mol/L), PPAR*δ* (GW501516; 0.1 *μ*mol/L) or PPAR*γ* (Rosiglitazone/BRL‐49653; Rosi; 1 *μ*mol/L). (D) With BSA alone (40 *μ*mol/L) or BSA‐FA (50 *μ*mol/L BSA‐oleic acid and 50 *μ*mol/L BSA‐linoleic acid) in combination with antagonists for PPAR*α* (GW6471; 0.1 or 1 *μ*mol/L), PPAR*δ* (GSK0660; 0.1 or 1 *μ*mol/L), or PPAR*γ* (GW9662; 0.1 or 1 *μ*mol/L) for 24 h. All wells had equal amounts of vehicle (0.1% DMSO) and BSA (40 *μ*mol/L). Relative mRNA expression levels of *Angptl4* were determined by quantitative RT‐PCR; All expression data were normalized to *Tbp*. Results are presented as means ± SD (*n* = 3, **P > 0.01) in C2C12 cells, and as means ± SEM in the murine experiment (4 control mice and 6 GW501516‐treated mice, *P > 0.05 and **P > 0.01). Student's *t*‐test was used for single comparisons.

Fatty acids and the PPAR*δ*‐specific activator GW501516 stimulate mRNA expression and secretion of ANGPTL4 in cultured myotubes (Staiger et al. 2009; Robciuc et al. 2012; Catoire et al. 2014). To provide physiological relevance to these in vitro findings, mice were gavaged with the PPAR*δ* agonist GW501516. This increased *Angptl4* mRNA threefold (Fig. 3A) and ninefold (Fig. 3B) in soleus and gastrocnemius muscle, respectively. To investigate if *Angptl4* was specifically induced by the PPAR*δ* isoform, C2C12 cells were transfected with PPAR*α*, PPAR*δ*, or PPAR*γ*, differentiated for 3 days and incubated for 24 h with PPAR isoform‐specific ligands (Fig. 3A). Expression of the different PPARs was similar after transfection (Bindesboll et al. 2013). Incubating the cells with ligands specific for PPAR*α* (WY‐14643), PPAR*δ* (GW501516), and PPAR*γ* (rosiglitazone), all enhanced expression of *Angptl4* mRNA (Fig. 3C). The PPAR*δ* ligand increased *Angptl4* mRNA about 11‐fold, whereas PPAR*α* and PPAR*γ* ligands both increased *Angptl4* expression about threefold. There was no effect on *Angptl4* transcription by transfecting the cells with the different PPARs. To investigate if FA‐induced *Angptl4* expression depended on a particular PPAR isoform, differentiated C2C12 cells were incubated with a combination of oleic acid and linoleic acid (50 *μ*mol/L each) in the presence of antagonists for PPAR*α* (GW6471), PPAR*δ* (GSK0660), and PPAR*γ* (GW9662) (Fig. 3D). FA‐induced expression of *Angptl4* was only significantly lower in the presence of the PPAR*δ* antagonist, which reduced the FFA‐induced *Angptl4* expression by approximately 70%. These results support that induction of *Angptl4* in vivo is mediated through FA activation of PPAR*δ*.

### Dexamethasone stimulates *ANGPTL4* expression in human myotubes

**Figure 4.**
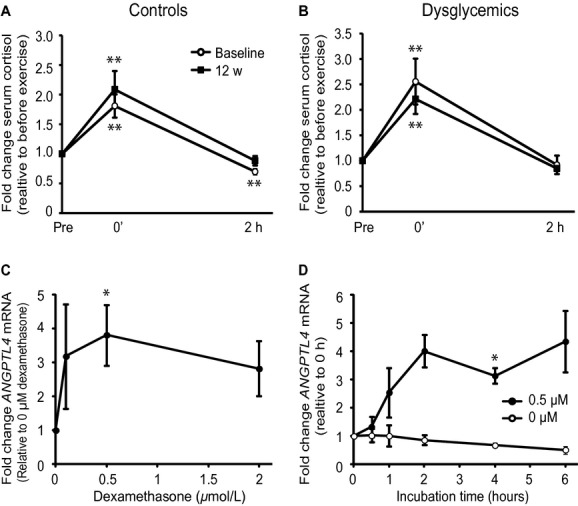
*ANGPTL4* is induced in human myotubes incubated with dexamethasone. Fold change in serum concentration of cortisol in (A) healthy (*n* = 13) and (B) dysglycemic men (*n* = 13) in response to acute exercise at baseline and after 12 weeks of training. Serum cortisol levels before (Pre) acute exercise were compared with immediately after (0′) 45‐min ergometer cycling (70% VO_2_max) and after 2‐h rest. (C) Primary human myotubes were differentiated for 6 days and incubated with 0, 0.1, 0.5, and 2 *μ*mol/L of dexamethasone for 2 h. Relative mRNA expression levels of *ANGPTL4* after 2 h of dexamethasone incubation were determined by quantitative RT‐PCR, presented as fold change relative to control (0 *μ*mol/L). (D) Myotubes differentiated for 6 days were incubated with 0.5 *μ*mol/L dexamethasone for 0, 0.5, 1, 2, 4, and 6 h. Fold changes in mRNA expression of *ANGPTL4* with and without dexamethasone after 0.5, 1, 2, 4, and 6 h were compared to zero time. All expression data were normalized to *RPLP0*. Data from three donors are presented as means ± SEM *P < 0.05 and **P < 0.01, Student's *t*‐test was used for single comparisons between matching time points.

Acute exercise elevates plasma levels of FFAs, which can activate PPAR*δ*. Plasma cortisol levels also increase in response to acute exercise (Kanaley et al. 2001). Cortisol can activate GR, another member of the nuclear receptor family, and expression of *ANGPTL4* is increased after GR activation in hepatocytes and adipocytes (Koliwad et al. 2009). In our study, serum concentration of cortisol was acutely increased between 1.8‐ and 2.5‐fold after 45‐min cycling, before as well as after 12 weeks of training in the controls (Fig. 4A) and dysglycemic subjects (Fig. 4B). There was no significant difference between the groups in exercise induction of circulating cortisol. Based on these observations, we investigated if activation of GR might regulate *ANGPTL4* gene expression in skeletal muscle cells by incubating primary human myotubes with the synthetic GR ligand, dexamethasone, in a dose–response experiment. Incubation of human myotubes with 0.5 *μ*mol/L dexamethasone for 2 h increased *ANGPTL4* expression 3.5‐fold (Fig. 4C). Myotubes were then incubated with 0.5 *μ*mol/L of dexamethasone up to 6 h. *ANGPTL4* mRNA level increased time dependently up to 2 h with dexamethasone compared to zero time (Fig. 4D). These results show that *ANGPTL4* can be regulated in vitro by the cortisol‐GR axis in addition to the FA‐PPAR*δ* axis, with possible implications for acute exercise.

### No effect of ionomycin or caffeine on *Angptl4* expression in cultured myotubes

**Figure 5.**
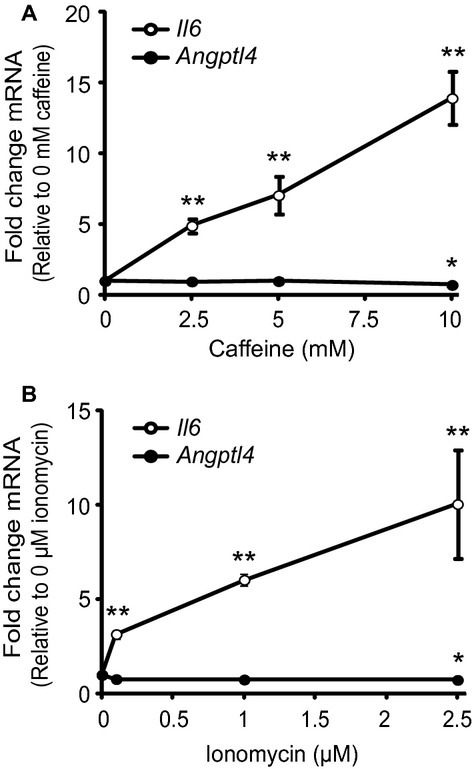
*Angptl4 *mRNA was unchanged in murine myotubes incubated with caffeine or ionomycin. (A) Differentiated C2C12 myotubes were incubated with caffeine (0, 2.5, 5, and 10 mmol/L) for 3 h. Relative mRNA expression levels of *Angptl4* and *Il6* were determined by quantitative RT‐PCR, presented as fold change relative to control (0 mmol/L caffeine). (B) Differentiated C2C12 myotubes were incubated with ionomycin (0, 0.1, 1, and 2.5 *μ*mol/L) for 3 h. Relative mRNA expression levels of *Angptl4* and *Il6* were determined by quantitative RT‐PCR, presented as fold change relative to control (0 *μ*mol/L ionomycin). All expression data were normalized to *Tbp*, and presented as means ± SD (*n* = 3, *P > 0.05, **P > 0.01). Student's *t*‐test was used for single comparisons.

Expression of *Angptl4* under different conditions was examined to address whether factors mimicking exercise in vitro also affected *Angptl4* expression. Neither caffeine nor inonomycin induced *Angptl4* mRNA (Fig. 5A and B); 10 mmol/L caffeine and 2.5 *μ*mol/L ionomycin even reduced *Angptl4* expression significantly in murine myotubes incubated for 3 h. Both caffeine and ionomycin had a dose‐dependent effect on mRNA expression of the positive control *Il6* (Fig. 5A and B). Caffeine and ionomycin had no dose‐dependent effect on *ANGPTL4* mRNA expression after 3 h and up to 24 h in human myotubes (data not shown).

### Acute exercise increased expression of murine *Angptl4* in muscle, adipose tissue, and liver

**Figure 6.**
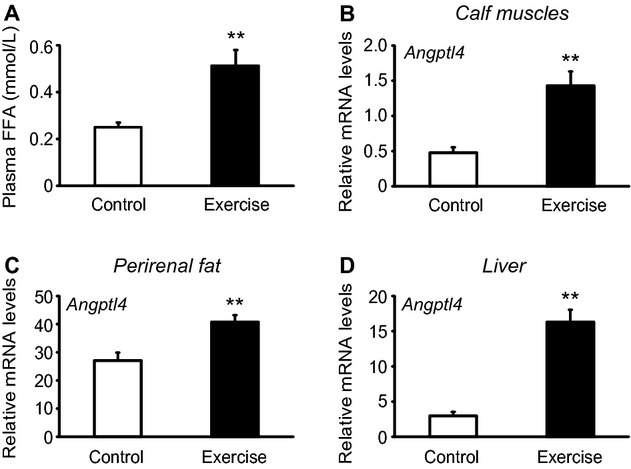
*Angptl4 *mRNA expression in muscle, fat, and liver increases after exercise in mice. Age‐ and weight‐matched C57Bl/6 mice underwent 60‐min treadmill exercise (*n* = 8), and the control mice rested in the cage (*n* = 8). Plasma samples and gastrocnemius muscles were collected immediately after exercise and in the control mice. The muscle biopsies were processed for mRNA expression analysis by quantitative RT‐PCR. (A) plasma levels of FFA; (B) *Angptl4 *mRNA in calf muscles; (C) *Angptl4 *mRNA in perirenal fat; (D) *Angptl4 *mRNA in liver. All expression data were normalized to *Tbp*. Bars depict means ± SEM **P < 0.01 between exercised mice and control mice. Student's *t*‐test was used for single comparisons.

It has been suggested that the liver is the main organ influencing the regulation of circulating ANGPTL4 (Dijk and Kersten 2014). To test if exercise induces *Angptl4* mRNA expression in other organs than skeletal muscle, we challenged mice with a treadmill run for 60 min or until exhaustion and harvested *m. gastrocnemius*, perirenal adipose tissue and liver. Mice were sacrificed immediately after treadmill exercise. Weight‐matched control mice (control mice: 37.9 ± 2.4 g, exercised mice: 37.9 ± 3.0 g) rested in the cage. We measured plasma concentration of FFAs (Fig. 6A) and mRNA levels of *Angptl4* in skeletal muscle (Fig. 6B), perirenal fat (Fig. 6C), and liver (Fig. 6D) in exercised and control mice. The exercised mice exhibited a twofold increase in plasma FFAs (Fig. 6A) and a threefold, 1.5‐fold and 5.5‐fold increase in *Angptl4* mRNA expression in skeletal muscle (Fig. 6B), perirenal fat (Fig. 6C), and liver (Fig. 6D) as compared to the controls, respectively. The mRNA level of *Angptl4* was higher in adipose tissue (controls: threshold cycle [*C*_t_] = 24.6 ± 0.5, exercise: *C*_t _= 24.2 ± 0.2) and liver (controls: *C*_t_ = 28.2 ± 0.9, exercise: *C*_t_ = 25.6 ± 0.5) than in skeletal muscle (controls: *C*_t_ = 30.1 ± 0.6, exercise: *C*_t_ = 28.4 ± 0.6). These data suggest that hepatic and adipose tissue may be more important contributors than skeletal muscle to circulating levels of Angptl4 both before and after exercise.

## Discussion

We have performed an exercise intervention study in normal weight controls and overweight dysglycemic subjects and seen that the mRNA level of *ANGPTL4* is increased in skeletal muscle during and after acute exercise which may be caused by increased levels FFAs as well as cortisol in the circulation. We also provide evidence suggesting that ANGPTL4 from muscle has limited impact on the serum level and show that muscle and adipose tissue contain two identical *ANGPTL4* mRNA isoforms.

One of the aims of this study was to investigate the contribution of skeletal muscle to the serum levels of ANGPTL4. Others have shown that muscle mRNA as well as fasting plasma levels of ANGPTL4 were enhanced in response to endurance exercise, especially after 4‐h recovery (Catoire et al. 2014). We also show that acute exercise increases ANGPTL4 mRNA levels in muscle and protein in serum. In addition, we now provide evidence suggesting that ANGPTL4 from muscle has a minor impact on the serum level compared to adipose tissue and the liver. Firstly, we show that the induction of *ANGPTL4* expression is more pronounced in skeletal muscle from exercising healthy controls as compared to overweight dysglycemic subjects, whereas the opposite is observed in serum, suggesting that other organs than skeletal muscle might be responsible for regulation of circulating ANGPTL4 in response to exercise. In mouse experiments, we demonstrate that *Angptl4* mRNA is induced in response to exercise in skeletal muscle, adipose tissue, and the liver, and show that liver and adipose tissue have a higher basal expression of *Angptl4* mRNA levels compared to skeletal muscle. Also, we show that the *Angptl4* mRNA induction in response to exercise is higher in the liver as compared to muscle. However, the fact that skeletal muscle accounts for about 40% of body mass in a lean individual might make it an important contributor to blood levels of ANGPTL4, although it has low *ANGPTL4* expression in muscle tissue. Thus, it is possible that skeletal muscle might be a more important source of circulating ANGPTL4 in subjects with more muscle mass and less adipose tissue. In our human exercise study, the group with the most adipose tissue exhibits the highest basal serum levels of ANGPTL4 and the highest induction of circulating ANGPTL4 in response to exercise. It has also been suggested that the liver is the main contributor to circulating ANGPTL4 during fasting (Dijk and Kersten 2014). Our data suggests that this is also the case in response to exercise.

The fact that during a one‐legged exercise intervention *ANGPTL4* mRNA expression was more induced in the resting leg as compared to the exercising leg suggests that circulating factors can induce ANGPTL4 transcription in skeletal muscle (Catoire et al. 2014). The plasma levels of FFA are known to increase during acute exercise and are hypothesized to increase transcription of ANGPTL4 in skeletal muscle after PPAR*δ* activation (Catoire et al. 2014). Studies on cultured human myotubes have revealed that secretion of ANGPTL4 is stimulated by FAs as well as a PPAR*δ*‐specific activator (Kersten et al. 2009; Staiger et al. 2009; Robciuc et al. 2012). In our study, we provide both in vitro and in vivo evidence for a robust increase of *Angptl4* transcription by PPAR*δ* in mouse skeletal muscle. We demonstrate by gavage feeding mice that a PPAR*δ*‐specific ligand enhances muscle *Angptl4* transcription in vivo. We also show that the FFA‐induced *Angptl4* expression was reduced in myotubes by an inhibitor specific for PPAR*δ*, but not by inhibitors directed against PPAR*α* and PPAR*γ*.

Several circulating factors are increased in response to acute exercise in addition to FFAs. One of the hormones that are known to be increased in response to exercise is the glucocorticoid cortisol (Nieman et al. 2005). Because it has been shown that ANGPTL4 is a direct GR target in hepatocytes as well as in adipocytes (Koliwad et al. 2009), one of the aims of this study was to investigate if human myotubes also respond to glucocorticoids by increasing *ANGPTL4* expression. We show that the serum concentration of cortisol is indeed increased in response to acute exercise. We also show that *ANGPTL4* transcription is elevated in cultures of primary human muscle cells incubated with the GR ligand, dexamethasone, comparable to the effects of dexamethasone on primary rat hepatocytes and human adipocytes (Koliwad et al. 2009). A recent study showed that circulating ANGPTL4 is not elevated by exercise when glucose is consumed (Catoire et al. 2014). Interestingly, both the plasma level of FFAs and cortisol are reduced when glucose is ingested during exercise (Nieman et al. 2005; Catoire et al. 2014) and may thus both explain some of the suppressive effects of glucose on circulating ANGPTL4 (Catoire et al. 2014). These results suggest that *ANGPTL4* mRNA in muscle can be regulated by the cortisol‐GR axis during acute exercise.

One function of ANGPTL4 may be to inhibit the uptake of FAs derived from lipoproteins by regulating LPL activity in skeletal muscle during exercise (Catoire et al. 2014). Interestingly, Catoire et al. (2014) suggested that during a one‐legged exercise intervention *ANGPTL4* mRNA expression was more increased in the resting leg as compared to the exercising leg because of the counteracting effect of AMPK on *ANGPTL4* transcription. The repression of ANGPTL4 production and hence enhanced LPL activity might promote use of circulating TG in the exercising muscle (Catoire et al. 2014). We show in our human exercise intervention that acute exercise induces both *LPL* and *ANGPTL4* transcription in skeletal muscle to a higher degree in control subjects as compared to the dysglycemic subjects. However, the serum levels of ANGPTL4 were more increased after exercise in the overweight dysglycemic subjects than in the controls. Because the relative contribution of skeletal muscle to circulating ANGPTL4 during exercise remains unknown, it is difficult to predict in which group ANGPTL4 has the largest inhibitory effect on LPL activity. Coexpression of *ANGPTL4* with *LPL* in skeletal muscle might suggest that ANGPTL4 acts mainly via local inhibition (Dijk and Kersten 2014). Future studies should address the relative contribution of skeletal muscle to circulating ANGPTL4. Thereby, it would be possible to evaluate the inhibitory potential ANGPTL4 may have on LPL activity during exercise.

In summary, our data suggest that ANGPTL4 produced in skeletal muscle during and after exercise has limited impact on the serum protein level and probably acts mostly in local tissue. Our data also indicate that FFAs and cortisol increase transcription of *ANGPTL4* in muscle during exercise via activation of PPAR*δ* and GR.

## Acknowledgements

We thank Hilde Nebb for access to experimental materials, Anne Randi Enget and Christin Zwafink for technical assistance, Harald Carlsen for donation of mice, Ansgar Heck and Birgitte Nellemann are for taking the biopsies, and Kristoffer J Kolnes, Daniel S Tangen, Tor I Gloppen, Torstein Dalen, Håvard Moen, Marius A Dahl, Guro Grøthe, Egil Johansen, Katrine A Krog, Øyvind Skattebo and Eirin N Rise for being responsible for and helping out on different aspects of the human strength and endurance intervention.

## Conflict of Interest

None declared.
